# Plate-based transfection and culturing technique for genetic manipulation of *Plasmodium falciparum*

**DOI:** 10.1186/1475-2875-11-22

**Published:** 2012-01-18

**Authors:** Florence Caro, Mathew G Miller, Joseph L DeRisi

**Affiliations:** 1 Department of Biochemistry and Biophysics, University of California San Francisco, San Francisco, California, USA; 2 Codexis, Inc., 200 Penobscot Drive, Redwood City, California 94063, USA; 3 Howard Hughes Medical Institute, University of California San Francisco, San Francisco, California, USA

**Keywords:** malaria, *Plasmodium falciparum*, transfection, 96-well plate

## Abstract

Genetic manipulation of malaria parasites remains an inefficient, time-consuming and resource-intensive process. Presented here is a set of methods for 96-well plate-based transfection and culture that improve the efficiency of genetic manipulation of *Plasmodium falciparum*. Compared to standard protocols plate-based transfection requires 20-fold less DNA, transient transfection efficiency achieved is approximately seven-fold higher, whilst stable transfection success rate is above 90%. Furthermore the utility of this set of protocols to generate a knockout of the PfRH3 pseudogene, screened by whole-cell PCR, is demonstrated. The methods and tools presented here will facilitate genome-scale genetic manipulation of *P. falciparum*.

## Background

Malaria remains a significant cause of human mortality with the vast majority of cases due to infection with *Plasmodium falciparum *[1]. Numerous efforts towards control and eradication of this disease are directed at different areas including insect vector control, vaccine development, and the discovery of new therapeutic drugs. All of these efforts benefit from a deeper understanding of the *Plasmodium *molecular biology.

Transgene expression or allelic exchange of altered or tagged versions of genes as well as knockouts are essential tools to study gene function and genetic interactions. In yeast, for example, a whole genome knockout and GFP tagged collection quickly followed the sequencing of the genome and led to an explosive growth of functional analysis and protein localization studies in this organism [2-4]. In *P. falciparum*, such tools would provide a wealth of information and a valuable resource to the community but technical hurdles remain.

Transient transfection of *P. falciparum *blood stages was reported 15 years ago [5] creating the possibility of transgene expression from an episomally maintained plasmid. Shortly thereafter stable transfection and homologous integration into the genome of drug-selectable constructs, was achieved [6]. Using genetically manipulated parasite lines, experiments including promoter analysis [7], localization studies [8], high-throughput drug screenings [9] and, importantly, the description of the first knockout [10] were possible, shedding light on important aspects of parasite biology, as well as its interaction with the human host and mosquito vector.

Gene knockouts are especially important for demonstrating essentiality of putative drug targets. Without genetic validation of drug targets, substantial resources may be wasted in the pursuit of inhibitors for non-essential gene products. However, since the complete genome sequence of *P. falciparum *became available [11], no significant shift from single-gene towards systematic whole-genome studies has occurred in this system. Only a small percentage (~2%) of the ~5,500 total putative coding regions have been disrupted and the largest scale study involved the targeting of 83 genes [12] reflecting the difficulty of genetically manipulating the parasite.

The process of creating a genetically modified *P. falciparum *cell line remains a cumbersome exercise. First, even though increasing the number of independent transfections can improve the chances of success, culturing *P. falciparum *is time consuming and resource intensive. Cultures need to be monitored daily and split manually using fresh human red blood cells (RBCs) suspended in relatively expensive media, making it difficult to simultaneously increase volume and carry a number of different cell lines. Second, the traditionally used transfection protocol [13] requires large amounts (50-100 ug) of plasmid construct and electroporation of each construct is performed one at a time in a single cuvette. Third, overall transfection efficiencies are low, 10^-2 ^to 10^-5 ^and 10^-6 ^for transient and stable transfections, respectively [8,14,15]. Finally, screening of successful transfections requires careful monitoring of resurgence of drug-resistant parasites by microscopic inspection. Integration of the plasmid construct, via single or double crossover homologous recombination, is rare and confirmation of the desired genetic modification by Southern Blot requires culture scale-up for the isolation of DNA.

**Figure 1:**
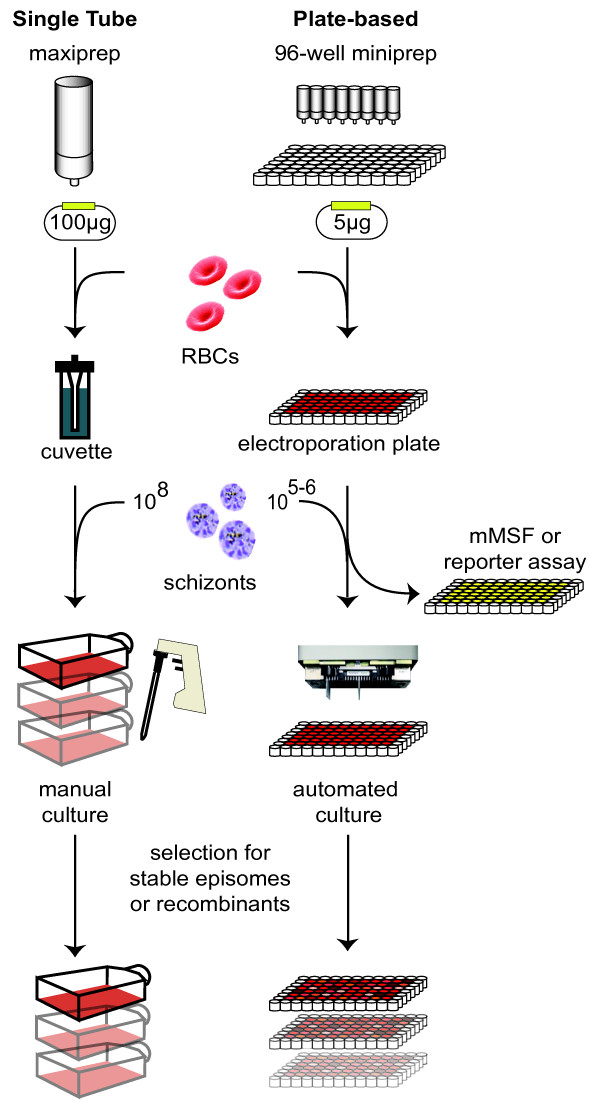
**Comparison of single cuvette and 96-well *Plasmodium falciparum *transfection methods**. In the traditional single-cuvette protocol (left), large amounts of a single plasmid construct are mixed with RBCs in a single cuvette for electroporation. Following addition of parasites, the culture is maintained manually in large flasks. In the plate-based method (right), up to 96 different plasmid constructs can be simultaneously transfected into RBCs using a 96-well electroporation plate. Parasites are added and the resulting cultures are maintained and monitored in 96-well format using a high-throughput liquid handler, without the need to scale up. Reporter assays, drug selection of parasites carrying stable episomes and generation of knockouts can all be efficiently performed in 96-well format.

Presented here is a transfection protocol and suite of methods for use with *P. falciparum*, with improvements over the traditional protocol at each step of the process (Figure 1). A major development is the adaptation of electroporation, culture maintenance, and monitoring in 96-well plate format for both transient and stable transfection experiments. Plate-based transfection requires 20-fold less plasmid DNA, yet results in an ~seven-fold increase in transient transfection efficiency and stable transfection success rates of > 90%. Using the optimized protocol gene knockouts were generated, screened, and validated entirely in 96-well format. The techniques developed here are anticipated to enable high-throughput genetic manipulation of *P. falciparum*, facilitating systematic, genome-wide studies of gene function and validation of putative drug targets.

## Methods

### Plasmid constructs

The pTI15 *Renilla *luciferase (RLUC) reporter plasmid was constructed by replacing 5'hrp3 for the ATP synthase (PFE0965c) promoter using the *KpnI *and *NsiI *sites on the pHRPGFPM2 vector [8]**(**MRA-69). RLUC was cloned into the *NsiI *and *XbaI *sites in place of gfpm2, with a 10 bp expression enhancing sequence (GTAAGTCAAA) immediately upstream of the start codon. PfRH3 knockout plasmids pHHT-TK-RH3KO and pCC1-RH3KO were constructed by amplification of the 5' and 3' homology region with primer pairs 5fw 5'-gcgCCGCGGaaagggttaatatataggacaattctttg-3', 5rv 5'-gcgACTAGTaggatcctctaaattgtgtatttca-3', and 3fw 5'-gcgGAATTCaatccgctgatagtactgaacagaaa-3', 3rv 5'-gcgCCATGGtcccatcaactaaggtttcatcatt-3', respectively. Digestion with *SacII*/*SpeI *and *EcoRI*/*NcoI *was followed by ligation of the amplified 5' and 3' homology regions, respectively.

### Parasite culture and transfection

200 μl culture volumes of *Plasmodium falciparum *W2 strain were carried in 96-well flat bottom plates at 4% hematocrit (HC). Fresh RBCs were collected from volunteer donors the day before, or the day of transfection, leukoreduced using the Baxter in-line filter and stored at 4°C until needed. Final transfection mixture was prepared at room temperature and consisted of 6 μl packed RBCs washed with 4°C RPMI 1640 with 0.2% sodium bicarbonate, 12.5 mM Na_2_ATP, 5 μg plasmid DNA (GeneElute HP Endotoxin-free plasmid Maxiprep kit, Sigma), 20 μl Amaxa SE solution resulting in a final volume of 32 μl. 20 μl of transfection mixture were transferred to the Amaxa Nucleocuvette plate (Lonza) and transfection was performed at room temperature. After applying the CM-162 pulse, the plate was incubated for 5 min at 37°C before addition of 80 μl of media warmed to 37°C. Cells were incubated for an additional 15 min at 37°C and transferred to a flat bottom 96-well culture plate containing 120 μl 37°C media. Electorporated RBCs were spun for 2 min at 90 xg. 180 μl of supernatant was removed and replaced with an equal volume of 37°C media. After incubation, 3-4 h at 37°C, MACS (Milteny Biotec) purified schizonts were added to eRBCs: 4 × 10^5 ^parasites for transient transfections and 1 × 10^6 ^for stable transfections. A detailed, step-by-step protocol for 96-well transfection is available on the DeRisi Lab website [16].

### Luciferase assay

48 h after RLUC reporter transfection, 180 μl of media was replaced with 180 μl of 0.003% saponin/PBS. 100 μl of the lysed culture was transferred to a 0.45 μm PVDF 96-well filter plate (Thomson Instruments) to harvest parasites by vacuum filtration. Retained parasites were washed with 200 μl 1X PBS and 30 μl 1X *Renilla *luciferase lysis buffer (Promega) was applied directly onto the filter surface. The filter plate was stacked on top of a white-well assay plate and incubated for 15 min. Parasite lysate was eluted by centrifugation at 913 xg for 5 min and luminescence was measured on a Veritas luminometer by injecting 100 μl of 1X *Renilla *luciferase assay reagent per well. A visual demonstration of the technique is available at [17].

### Stable transfection

Cultures were split to a final 0.5% HC 24 h after electroporation. At 48 h post-electroporation, fresh RBCs were added to a final 2% HC with media containing either 0.5 mg/ml G418 (Geneticin, GIBCO) or 2.5 nM WR99210 for positive selection. Thereafter, drug-containing media was exchanged daily. At day 8 post-electroporation, RBCs were added to a final 4% HC. Thereafter, cultures were maintained by weekly alternating two treatments, addition of 0.5% HC and replacement of 30% of the culture, each performed once and separated by three or four days, until any parasitaemia was detected. For selection of knockouts, negative selection was applied once cultures reached at least 1% parasitaemia, using either 10 μM Ganciclovir (Cytovene, Roche) or 1 μM 5-FC (Sigma). Clonal isolates originating from a single parasite were obtained by limiting dilution at 0.3 parasites/well at 4% HC.

### Modified MSF assay

The malaria SYBR green assay was performed according to Smilkstein *et al *[18], with modifications. Following a media change, 10 μl of resuspended culture was added to a mixture of 10 μl 1X PBS and 20 μl MSF lysis buffer with 1X SYBR Green I (Molecular Probes) in Corning 384-well black assay plates. Signal was measured using the Analyst HT (Molecular Devices) with 485 nm excitation, 530 nm emission and 505 nm dichroic, for 40 msec per well with a 1% neutral density filter to allow linear signal acquisition on the SmartRead 2 setting.

### *Plasmodium falciparum *whole-cell PCR

20 μl aliquots were taken from cultures at 0.1-10% parasitaemia and 4% HC. Pellets were flash-frozen in liquid nitrogen (suitable for storage at -80°C), then thawed by addition of 40 μl ice-cold H_2_O. 10 μl of lysed cells were quickly added to 10 μl of PCR mixture on ice to yield final concentrations of 1X Phusion HF Buffer (Finnzymes), 0.5 μM each primer 320 μM each dATP and dTTP, 80 μM each dCTP and dGTP, and 0.8U Phusion polymerase (Finnzymes). Two-step cycling parameters were 98°C (2 min); 35 cycles of 98°C (10 sec), 60°C (1 min/kb); final extension at 60°C (4 min). Primers used to detect the integration junction were, ko5'-fw 5'-ttttggtttacacattttgagatga-3', ko5'rv, 5'-ttaccttctactgaagaggttgtgg-3', ko3'-fw, 5'- ttcatgttttgtaatttatgggatagc -3' and ko3'-rv 5'-ctgtagcaacatcagaacttggtgtat-3'. Internal ORF fragment detection primers used were wt-fw 5'-gaacaatgtacaagatgtgtatgatgaga-3' and wt-rv 5'-acacttttcagctgcctctaatgttat-3'

### Southern blots

Primer pairs 5fw/5rv and 3fw/3rv were used to generate 5' and 3' probes respectively. 3 μg of genomic DNA was digested either with *SphI/PvuII *or *BglII *for the 5' probe, or with *SphI*/*EcoRI *or *SpeI*/*PvuII *for the 3' probe, separated on a 1% (w/v) agarose gel and transferred to Hybond N+ membrane (Amersham) according to the manufacturer's instructions. Radio-labelled probes were generated using the Prime-It II Random Primer Labeling Kit (Stratagene). Hybridization signals were detected with PhosphorImager analysis (Molecular Dynamics).

### Automation

Culture manipulations, MSF assays, blood smears, cryopreservation, and thawing of cultures were performed using a Beckman 96-channel BioMek-NX liquid handler inside a Baker BioProtect II biosafety cabinet paired to a Thermo-Fisher Cytomat automated combination plate server and mixed gas incubator. 96-well blood smears were performed on custom 12.7 × 8.5 cm slides (Thermo Fisher Scientific). A video of the process is available at [19].

## Results

### Optimization of plate-based electroporation conditions with a luciferase reporter assay

For 96-well transfection, the Amaxa Nucleofector 96-well shuttle platform (Lonza) was chosen based on reports that a related single-cuvette device improved the transfection efficiency of *Plasmodium berghei *by 1,000-fold [20,21].

Transfection efficiency is extremely sensitive to small variations in parameter settings: Insufficient voltage incompletely permeabilizes cell membranes reducing transfection efficiency and excess voltage can lead to irreversible cell damage. Moreover, the precise transmembrane voltages achieved are critically dependent on salt concentrations and buffer conditions. Optimizing transfection conditions therefore requires probing an extensive matrix of parameters. For rapid and simultaneous measurement of different electroporation conditions, a plate-based *Renilla *luciferase (RLUC) reporter assay was developed.

First, to validate the ability of the RLUC assay to assess differences in transfection efficiency, a detailed characterization of three potential sources of noise within the system was performed: luminometer reading, the RLUC assay, and the transfection. As expected, the noise introduced by the luminometer reading was the smallest (CV = 10%) whereas the variation observed for the RLUC assay and among individual replicates of transfections was moderately larger (CV = 24% and CV = 23%, respectively) (Additional file 1). Thus, with typical signals 400-fold higher than the background, the RLUC reporter assay allows rapid and functionally accurate measurement of transfection efficiencies.

Using this assay, electroporetic pulse and buffer combinations, cell volume, and other transfection mixture components were optimized. In addition, the minimal amount of DNA sufficient for efficient transfection was determined.

**Figure 2:**
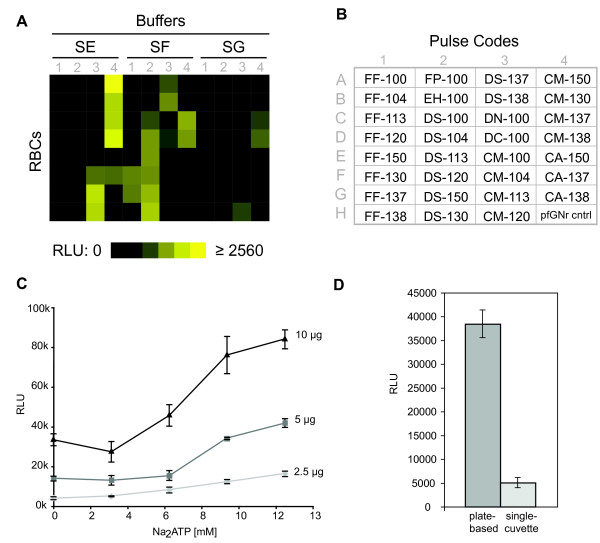
**Reporter signal increases with both DNA and Na**_**2**_**ATP concentration**. (A) RBCs were transfected in a 96-well electroporation plate with 31 different pulses, in the three different buffers, SE, SF and SG. RLUC reporter activity in each well of the plate is shown as an intensity heatmap. (B) Set of pulses tested and their corresponding position on the electroporation plate. Position H4 is the pfGNr transfected negative control (pulse CM-150) (C) Increasing amounts of RLUC reporter plasmid DNA (pTI15) (2.5 μg, light gray diamond; 5 μg, dark gray square; 10 μg, black triangle) and increasing concentrations of Na_2_ATP (0, 3, 6, 9, 12.5 mM) in SE buffer were mixed with 6 μl packed RBCs. Four independent transfection wells were assembled for each condition. *Renilla *luciferase activity was measured 48 h post-transfection. RLU, relative luminescence units. Error bars, s.e.m. (D) Transfection efficiency of single cuvette and plate-based methods. For the single cuvette transfection, the previously published protocol was followed [13]. The plate-based transfection was performed using the optimized protocol reported here (see Methods). Late stage parasites were added to pooled, single cuvette or 96-well, eRBCs and the mixture was re-split into four wells of a flat bottom 96-well culture plate eliminating outgrowth differences. Luciferase activity was measured in the four culture wells, normalizing for the number of parasites. Error bars, s.d.

Selection of the optimal pulse-buffer combination was carried out by electroporation of the RLUC reporter plasmid into RBCs in each of three buffers provided by the manufacturer, followed by addition of parasites and measurement of RLUC signal after 48 h (Figure 2A and 2B). For each buffer, 31 different instrument pulse settings were tested. The highest luciferase signals were obtained with buffer SE and pulses in the CM series. A second round of optimization using eight additional pulses with buffer SE was conducted and pulse "CM-162" was found to provide a combination of high transfection efficiency with moderately low RBC lysis following electroporation (Additional file 2). Electroporation of parasite-infected RBCs yielded comparable results (Additional file 3) but required significantly more preparation. The RBC loading strategy was chosen for all subsequent transfections due to ease and flexibility of handling these cells.

Intraerythrocytic levels of ATP are critical to support parasite development and can be depleted via leakage from permeabilized membranes during the electroporation process [22]. Therefore, the effect of Na_2_ATP addition on transfection efficiency was tested. Varying amounts of the RLUC reporter plasmid were transfected with increasing concentrations of Na_2_ATP into a constant volume of packed RBCs. A five- and four-fold increase in luciferase activity was observed at the highest concentrations of DNA and Na_2_ATP tested (Figure 2C). Importantly, this effect is not due to the use of ATP in the bioluminescent reaction, since *Renilla *luciferase catalyzes the production of light in the absence of ATP, requiring only the corresponding substrate and oxygen. Repeating the experiment with Li_2_ATP and MgATP yielded values two- and five-fold lower than those obtained using Na_2_ATP, respectively (Additional file 4A). Furthermore, varying the volume of packed RBCs in the mixture showed no luciferase activity enhancement (Additional file 4B).

Based on these data, we chose 6 μl of packed RBCs, 5 μg of plasmid DNA and 12.5 mM Na_2_ATP as standard operating conditions for subsequent transient and stable transfections. Although overall reporter activity of cells electroporated with 10 μg of plasmid was approximately two-fold higher, a lower amount of plasmid DNA for subsequent experiments was chosen for compatibility with plate-based systems for plasmid purification. This amount of DNA is 20-fold less than called for by most commonly used *Plasmodium falciparum *transfection protocols.

To assess the operational efficiency of the optimized protocol, we compared it to a standard, RBC-loading, single electroporation cuvette protocol [13]. After electroporation using both methods, eRBCs (electroporated RBCs) were plated at an equal haematocrit (percentage of total volume occupied by RBCs) into the wells of a 96-well flat-bottom plate and inoculated with the same number of late stage parasites for equal outgrowth conditions. The efficiency of the plate-based method was almost eight-fold greater than the single cuvette protocol, on a per-parasite basis (Figure 2D). The protocol presented here facilitates high-throughput transfection of up to 96 plasmids in parallel with higher efficiencies and reduced reagent requirements as compared to single-cuvette transfection.

### Automated continuous culture

Simultaneous culture of hundreds of *Plasmodium falciparum *strains is challenging using traditional methods such as individual microscopic inspection of blood smears, and manual media change and subculture. Therefore automated protocols for maintenance and parasitaemia quantification of microliter-volume cultures in 96-well plates using an integrated liquid handler/incubator system were developed.

Insufficiently dense cultures can adversely affect culture growth by hindering parasite propagation after RBC egress, while too-dense cultures deplete nutrients within media too quickly. Thus, the impact of cell density on culture health of cultures grown in plates at different hematocrits was measured. The growth rate of the parasites was modestly affected by the range of haematocrit percent tested (Additional file 5), with an optimum at 4% haematocrit.

Commonly implemented methods for the rapid and quantitative measurement of parasitaemia are flow cytometry [23], plate-based fluorescence assays [18], or enzyme driven colorimetric assays [24]. In this work, a modified and automated malaria SYBR Green I fluorescence (mMSF) assay [18], where fluorescence signal is proportional to parasitaemia, for use with as little as 10 μl culture volumes in 384-well plates, was developed. The background fluorescence in infected or uninfected RBC-containing wells was found to be higher in spent culture media as compared to freshly prepared media (Additional file 6). By changing the media prior to the assay and washing with PBS prior to the addition of lysis buffer the signal to background (infected vs. uninfected RBCs) increased by approximately five-fold. For a parasitaemia of 4%, this corresponds to a signal to background ratio of 20:1. Further rinsing with PBS produced additional gains in the signal to background ratio, although this is at the expense of added time and effort.

The mMSF assay provides quantitative information about levels of parasitaemia but it cannot provide qualitative information about parasite health and morphological stage. Manual inspection of Giemsa-stained blood smears provides both quantitative measurements of parasitaemia and information detailing parasite health and morphological stage. However, traditional production and inspection of blood smears is labour-intensive and time consuming. For this purpose a 96-well automated blood smear protocol, whereby 1.5 μl of each culture well is simultaneously spread over a 0.45 cm^2 ^area on a custom large-format slide, was developed. The smears from this assay are qualitatively similar to standard blood smears and can be coupled to previously published image-processing algorithms [25,26] to completely automate the process of determining levels of parasitaemia from thin smears (Additional file 7).

Both the mMSF assay and the automation of parasite imaging using Giemsa-stained blood smears provide an important set of tools compatible with the high-throughput transfection and culture procedures described above.

### High-throughput stable transfections

To test whether the chosen standard operating conditions for transient transfection were also suitable for the generation of stable episomes, three different plasmids were used: pfGNr, an episomal GFP expression vector [8], and pHHT-TK [27] and pCC1 [28], two commonly used vectors for the generation of knockouts by double crossover recombination.

**Figure 3:**
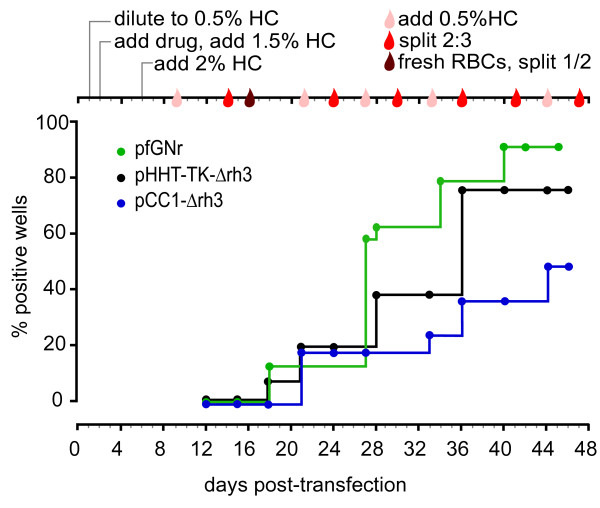
**High-throughput stable transfection**. Three plasmids, pfGNr (green), pHHT-TK-Δrh3 (black), pCC1-Δrh3 (blue) were transfected using the optimized protocol (see Methods). Dots on the growth line represent days at which wells were screened for parasites either through fluorescence microscopy (pfGNr) or Giemsa-stained smears (pfGNr, pCC1-Δrh3 and pHHT-TK-Δrh3). Schedule of outgrowth conditions consists of weekly blood additions (0.5% HC, light red drop) and 2:3 culture splits (red drop). Note a large split of the culture at two weeks post-transfection. Data are shown as percentage positive wells where the detection of a ring in a culture well equals a positive well and 24 and 16 are the total number of wells for pfGNr and for pCC1 and pHHT-TK, respectively. Day 0 = transfection day.

Twenty-four independent, plate-based transfections of pfGNr were set up using the optimized protocol. The resulting culture wells were put under drug pressure 48 h after transfection to select for cells carrying the plasmid with the drug resistant marker (Figure 3). By day 18 post-electroporation, drug-resistant parasites were detected for three of 24 wells by blood smear inspection and fluorescence microscopy for GFP detection. This number steadily increased over the next three weeks to 22 of 24 (> 90%) wells containing live, GFP-positive parasites by week 6 post-electroporation.

In parallel, 16 independent, plate-based transfections using the knockout vectors pHHT-TK and pCC1, were performed. The electroporated cultures were maintained on an identical schedule of outgrowth to that of the pfGNr transfection experiment. The first live parasites were visible on day 18 and day 21 for pHHT-TK and pCC1, respectively. By day 46 post-electroporation, 75% (12 of 16) of the pHHT-TK and 50% (eight of 16) of the pCC1 culture wells contained drug-resistant parasites detected by blood smear inspection.

These results demonstrate that stable transfection of a variety of commonly used vectors can be efficiently achieved using the optimized, plate-based transfection protocol, with the benefit that selection, culture maintenance and monitoring can all be performed in 96-well format.

### PfRH3 knockout in plate format

The next step to generating a knockout is the selection of parasites with chromosomally integrated copies of the plasmid away from those carrying the episomal forms. This can be achieved by the incorporation of negative selection markers on the knockout plasmids whose gene product converts a normally innocuous metabolite into a toxic one. Cells that do not express the negative selection marker and contain the desired integration event are enriched by simultaneous application of positive and negative selection pressure.

**Figure 4:**
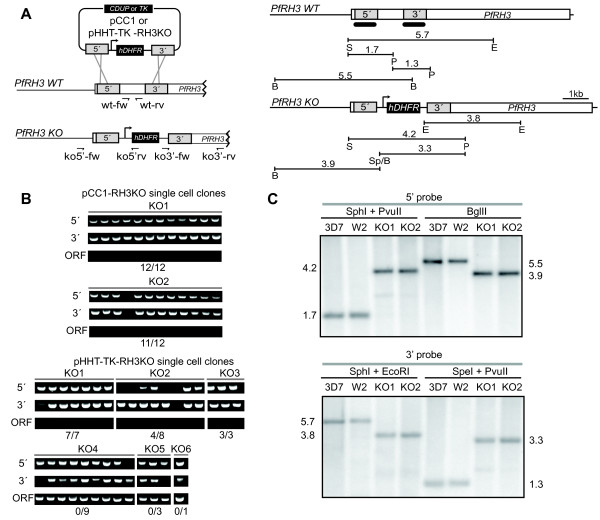
**High-throughput gene knockout and validation**. (A) Schematic representation of the recombination between the pCC1 or pHHT-TK-RH3KO plasmids and the PfRH3 target locus. Primers used to detect 5' (ko5'-fw and rv), 3' (ko3'-fw and rv) loop-ins and WT ORF (wt-fw and rv) are depicted as small arrows. Restriction fragment lengths are shown to scale. 5' and 3' probes are represented as two thick bars. (B) Whole-cell PCR on single cell clones of putative knockouts. Lanes show products for the 5', 3' loop-ins and WT ORF for each individual clonal isolate. Upper panels, pCC1-Δrh3 clonal isolates derived from two stably transfected culture wells (KO1 and 2). Lower panels, pHHT-TK-Δrh3 clonal isolates derived from six stably transfected culture wells (KO1-6). (C) Southern blot of genomic DNA from 3D7 WT strain, parent W2 WT strain, and both pCC1 KO1 and KO2 generated strains.

As a test case for performing the entire protocol through generation of a knockout, short homologous sequences (~1 kb) to the coding region of the non-essential pseudogene PfRH3 [29] were cloned into the pCC1 (pCC1-Δrh3) and pHHT-TK (pHHT-TK-Δrh3) plasmids (Figure 4A) for double-crossover recombination and knockout of this locus. These knockout vectors only differ in their negative selection markers, *Herpes simplex *virus thymidine kinase gene (HSV-tk) [27] and the *Saccharomyces cerevisiae *cytosine deaminase/uracil phosphoribosyl transferase (*Sc*CDUP) (*FCY1*) gene [30] in pHHT-TK and pCC1, respectively.

After stable transfection of the knockout vectors was successfully achieved (see above) six of the pCC1-Δrh3 and 10 of the pHHT-TK-Δrh3 tranfected culture wells under positive selection were subjected to negative selection to enrich for putative integration events. For all of them a fraction of the culture was kept in a parallel plate under positive selection only. After applying negative selection, parasitaemia declined to undetectable levels over seven days. Ring-stage parasites were detectable for six of the 10 pHHT-TK-Δrh3 cultures 17 days after the application of double selection. No parasites emerged from the pCC1-Δrh3 wells from this round of double selection. Therefore, three concentrations of the negative selection drug (5-FC: 1, 0.3, 0.1 μM) were applied to the parallel culture fraction grown an additional 12 days under positive selection only. After approximately two weeks under double selection, parasites were visible for two of the six culture wells at all 5-FC concentrations. These results suggest that culturing parasites for an additional period before applying negative selection may be beneficial in this plate-based format.

The Southern blot technique has traditionally been used for confirmation of knockouts [31], but requires large amounts of genomic DNA, making it difficult to adapt to a high-throughput transfection pipeline. Therefore, a plate-based whole-cell PCR protocol was developed to rapidly screen for putative knockout cultures prior to confirmation by Southern Blot. To eliminate spurious cultures resulting from analysis of mixed populations containing both transfected and untransfected parasites, populations were plated at limiting dilution to obtain clonal isolates. Whole-cell PCR was performed on 12 clonal isolates of each of the populations to detect the 5' and 3' integration junctions and the internal wild-type (WT) ORF fragment (Figure 4B). All clones except for one yielded the correct PCR results, indicating integration of the plasmid by double-crossover recombination and deletion of the target locus. A traditional Southern blot analysis revealed that the two original parasite populations were in fact composed of true knockouts (Figure 4C).

Previous work has found that spontaneous resistance to negative selection using the TK marker in pHHT-TK is common and gives rise to ganciclovir-resistant cultures [28,30]. Consistent with these reports, of all pHHT-TK-Δrh3 transfected parasites populations growing on double selection (KO1-6), only two yielded true knockout clones (KO1 and 3) and one showed correct PCR results for half of the clones (KO2). The remaining three candidates yielded no correct clones (KO4-6). It is speculated that in some of the latter cases, single crossover disruption, rather than double crossover knockout, of the locus occurred, because 5' and 3' integration junctions as well as the internal wild-type ORF fragment could be detected. These results indicate that the ScCDUP negative selection marker present on the pCC1 plasmid is superior based on the proportion of true knockout clones obtained.

In all, using our plate-based transfection protocol multiple clonal PfRH3 knockouts were successfully obtained, rapidly screened by a robust high-throughput whole-cell PCR assay and ultimately confirmed by Southern Blot.

## Conclusion

The suite of techniques developed here enables high-throughput transfection in *Plasmodium falciparum *by means of parallelization and automation. The core of this suite is parasite transfection, selection, and maintenance, all executed entirely in a 96-well plate format. While requiring 20-fold less DNA, 20-fold fewer RBCs and, 100-fold fewer parasites than the traditional transfection protocol, transient transfection efficiency was almost eight-fold higher on a per-parasite basis. Furthermore, > 90% success in generating stably transfected cultures using an episomal GFP reporter or 50-75% success with knockout vectors was shown and these cultures were carried forward to generate knockouts.

The general approach also relies on whole-cell PCR as an efficient alternative for screening of a large number of putative knockouts, whereby true knockouts can be rapidly differentiated from those where the wild-type version of the target gene is still detectable. A major advantage of the whole-cell PCR technique is that it does not require the scale-up of cultures beyond the volume of a well in a 96-well plate, nor does it require DNA isolation steps. The positively screened knockouts can be then scaled-up and further analyzed and confirmed using conventional Southern Blot.

The methods presented here are amenable to further improvements in automation. In particular, the requirement for production of single cell clones by limiting dilution is inherently inefficient and leads to a large expansion of culture plates. To avoid manual and laborious alternatives, such as micromanipulation with a glass needle, it is possible to use microfluidic dielectrophoresis sorting strategies to automatically seed wells with a single infected RBC as recently described by Miao *et al *(2010) [32]. Also, the generation of stably transfected lines is still required for generating recombinants and there is no circumvention of the long periods of culture involved in the process. Furthermore, low recombination efficiencies may be overcome by using an integrase-mediated site specific recombination system as the one described [33]. Future advances to improve these aspects of the protocol could greatly facilitate genetics by homologous recombination in *P. falciparum*.

Irrespective of the challenges that lie ahead, conducting *P. falciparum *transfections in 96-well plates represents an improvement over the traditional one at a time method in resources, throughput, and time, increasing the chances of success by increased efficiencies and parallelization.

## Competing interests

The authors declare that they have no competing interests.

## Authors' contributions

FC designed and performed experiments, analyzed data and wrote the paper. MM designed and performed experiments. JDR conceived of the study, and participated in its design, coordination, data analysis, co-wrote the manuscript, and co-designed the figures. All authors read and approved the final manuscript.

## Supplementary Material

Additional file 1 **RLUC assay noise measurements**. Boxplots of normalized RLU values for each of the noise measurements. Transfection noise was measured on 44 independently transfected and cultured wells. Assay noise was measured by pooling and re-splitting 40 transfection wells eliminating transfection noise. Luminometer reading noise was measured by pooling and re-splitting the lysate of 40 transfection wells prior to addition of RLUC substrate, eliminating transfection noise and assay noise. In each case, four wells of negative control plasmid, pfGNr, were transfected in parallel (bckg = background). Click here for file

Additional file 2 **Transient transfection with an extended set of pulses**. 6 μl of packed RBCs were transfected with 5 μg RLUC reporter plasmid, 12 mM Li_2_ATP and Buffer SE using pulses in the CM series and eight additional ones. Four replicas of each condition were setup. HC was determined measuring absorbance at 410 nm and normalizing to a standard curve of non-pulsed RBCs at known HC. In bold is the chosen pulse, CM-162. ± s.d., standard deviation. Click here for file

Additional file 3 **Optimizing buffer and pulse codes for transient transfection in plates**. (A) Set of the 31 pulses tested and their corresponding position on the electroporation plate. (B) Intensity heatmaps of RLUC reporter activity for direct ring and schizont electroporation. Transfection procedure: 4 ul packed RBCs or 6% ring or schizont stage parasites, were mixed with 5 μg of the RLUC reporter plasmid in one of three buffers (SE, SF, or SG). Each of the 31 pulses was delivered to the corresponding wells. pfGNr was used as negative control. Luciferase activity was measured 48 h and 72 h after electroporation, for RBC or schizonts, and for rings, respectively. In parallel with the ring and schizont transfections, RBCs were electroporated as in B using pulse CM-150 and buffer SE. Click here for file

Additional file 4 **Optimization transfection mixture components**. (A) Transfection efficiency using different ATP salts. 6 μl packed RBCs were transfected (pulse CM-162) with 5 μg of the RLUC reporter plasmid, in Buffer SE and 6.25 mM final concentration of different ATP salts (Na_2_ATP, Li_2_ATP and MgATP). RLUC reporter signal was measured 48 h later for six replicas of each condition. Error bars, s.e.m. (B) Transfection efficiency using different volumes of packed RBCs. Three different volumes (2, 4 and 6 μl) of packed RBCs were transfected (pulse CM-162) with 2.5, 5 or 10 μg RLUC reporter plasmid, in Buffer SE with or without 10 mM Li_2_ATP. Table shows RLU values measured 48 h later for two replicas of each condition. Click here for file

Additional file 5 **Optimization of hematocrit for culture in 96-well plates**. 200 μl of 0.025, 0.013, 0.006, 0.003% parasitaemia cultures (ring stage) were plated at 1, 2, 3, 4, and 5% HC in a 96-well flat-bottom plate, in triplicate. Five days later parasitaemia was measured by mMSF assay for each condition. Error bars, standard deviation. Click here for file

Additional file 6 **Modified MSF assay**. (A) For each tested condition, 200 μl of 0.8% parasitaemia cultures at 2% HC, were set up in columns 1 to 10 of five 96-well plates, uninfected RBCs were plated in column 11 for background signal measurement, and column 12 was used to generate a standard curve for parasitaemia. Final parasitaemia was measured 72 h later by MSF assay using five different manipulations before the measurement; **media change**, spent media was replaced with fresh media; **no media change**, culture was resuspended in the spent media; **media wash/PBS resuspend**, spent media was replaced with fresh media and culture was washed with 1X PBS; **PBS resuspend**, spent media was replaced with 1X PBS; **PBS washed**, spent media was replaced with 1X PBS and further washed in PBS. (B) For each of the five manipulations the mean of 10 column measurements (n = 80), their corresponding mean background signals (n = 8) and signal to background ratios, are listed. (C) Standard curve obtained using the PBS resuspend manipulation. Note that parasitaemias as low as 0.5% can be detected. Click here for file

Additional file 7 **Automated blood smearing**. (A) Custom slide dimensions. (B) Snapshot of the automated blood smearing process. The Beckman 96-channel BioMek-NX liquid handler dispenses 1 μl of culture at 0.1 mm above the slide surface as the tips trace two concentric 0.33 and 0.66 mm wide squares. (D) Representative Giemsa-stain of one of the 96 smears on a slide [19]. Click here for file
