# Supplementary material for: Plate-based transfection and culturing technique for genetic manipulation of Plasmodium falciparum
Source: Malar J. 2012 Jan 18;11:22. doi: 10.1186/1475-2875-11-22 (PMC3293776; doi:10.1186/1475-2875-11-22)
Supplement: Additional file 1 — RLUC assay noise measurements. Boxplots of normalized RLU values for each of the noise measurements. Transfection noise was measured on 44 independently transfected and cultured wells. Assay noise was measured by pooling and re-splitting 40 transfection wells eliminating transfection noise. Luminometer reading noise was measured by pooling and re-splitting the lysate of 40 transfection wells prior to addition of RLUC substrate, eliminating transfection noise and assay noise. In each case, four wells of negative control plasmid, pfGNr, were transfected in parallel (bckg = background). [file 1475-2875-11-22-S1.PDF]

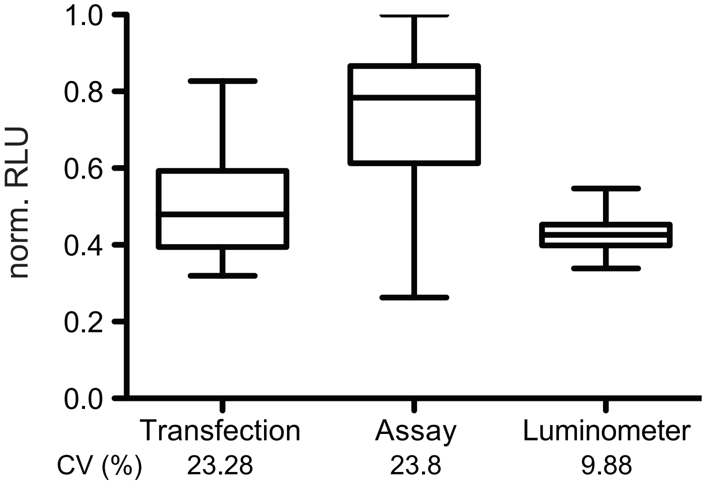

	Transfection		Assay		Luminometer	
normalized RLU	data	bckg	data	bckg	data	bckg
Mean	0.50	9.75×10^{-4}	0.74	1.01×10^{-3}	0.43	1.06×10^{-3}
Std. Deviation	0.12	2.24×10^{-4}	0.17	7.69×10^{-5}	0.04	4.05×10^{-5}
Std. Error	0.02	1.12×10^{-4}	0.03	3.85×10^{-5}	0.01	2.02×10^{-5}
