# Supplementary material for: Plate-based transfection and culturing technique for genetic manipulation of Plasmodium falciparum
Source: Malar J. 2012 Jan 18;11:22. doi: 10.1186/1475-2875-11-22 (PMC3293776; doi:10.1186/1475-2875-11-22)
Supplement: Additional file 2 — Transient transfection with an extended set of pulses. 6 μl of packed RBCs were transfected with 5 μg RLUC reporter plasmid, 12 mM Li2ATP and Buffer SE using pulses in the CM series and eight additional ones. Four replicas of each condition were setup. HC was determined measuring absorbance at 410 nm and normalizing to a standard curve of non-pulsed RBCs at known HC. In bold is the chosen pulse, CM-162. ± s.d., standard deviation. [file 1475-2875-11-22-S2.PDF]

	Pulse Code	Luminescence (RLU x10^3 ± s.d.)	Hematocrit after pulse (% ± s.d.)
Selected pulses	CM - 130	127 ± 10	1.13 ± 0.09
	CM - 138	120 ± 25	1.07 ± 0.07
	CM - 150	119 ± 8	1.12 ± 0.03
	CM - 137	89 ± 8	0.67 ± 0.06
	CM - 120	84 ± 16	1.32 ± 0.05
	CM - 113	77 ± 14	1.34 ± 0.09
Extended Pulse Set	CM - 119	126 ± 22	1.22 ± 0.08
	**CM - 162**	**126** ± **17**	**1.13** ± **0.07**
	CM - 151	122 ± 5	0.71 ± 0.01
	CV - 150	110 ± 26	0.88 ± 0.05
	CV - 151	106 ± 12	1.11 ± 0.10
	CM - 145	88 ± 29	1.24 ± 0.12
	CV - 138	82 ± 12	0.62 ± 0.04
	CV - 120	76 ± 17	0.94 ± 0.04
	No pulse	0.073 ± 0.002	1.83 ± 0.04
