# Supplementary material for: Plate-based transfection and culturing technique for genetic manipulation of Plasmodium falciparum
Source: Malar J. 2012 Jan 18;11:22. doi: 10.1186/1475-2875-11-22 (PMC3293776; doi:10.1186/1475-2875-11-22)

A

Pulse Codes

	1	2	3	4
A	FF-100	FP-100	DS-137	CM-150
B	FF-104	EH-100	DS-138	CM-130
C	FF-113	DS-100	DN-100	CM-137
D	FF-120	DS-104	DC-100	CM-138
E	FF-150	DS-113	CM-100	CA-150
F	FF-130	DS-120	CM-104	CA-137
G	FF-137	DS-150	CM-113	CA-138
H	FF-138	DS-130	CM-120	pfGNr cntrl

B

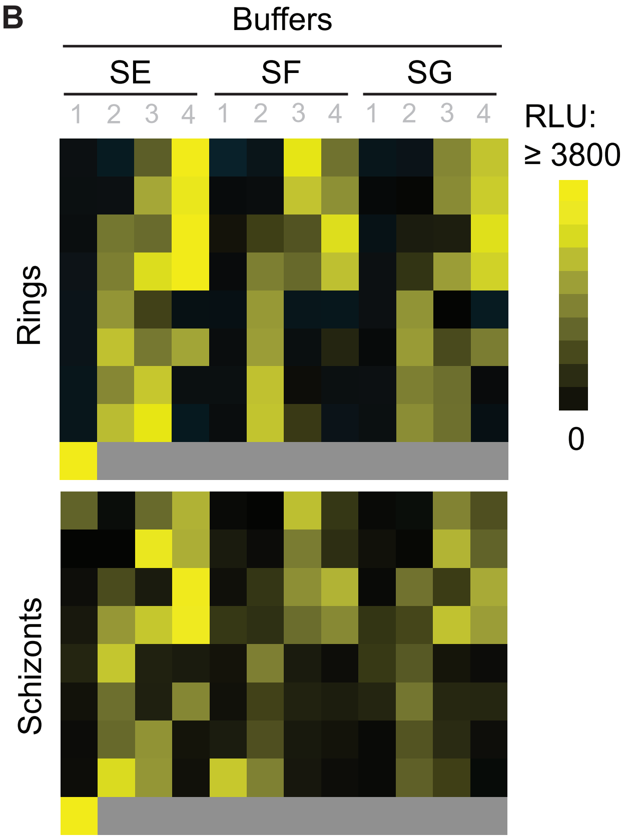

Supplement: Additional file 3 — Optimizing buffer and pulse codes for transient transfection in plates. (A) Set of the 31 pulses tested and their corresponding position on the electroporation plate. (B) Intensity heatmaps of RLUC reporter activity for direct ring and schizont electroporation. Transfection procedure: 4 ul packed RBCs or 6% ring or schizont stage parasites, were mixed with 5 μg of the RLUC reporter plasmid in one of three buffers (SE, SF, or SG). Each of the 31 pulses was delivered to the corresponding wells. pfGNr was used as negative control. Luciferase activity was measured 48 h and 72 h after electroporation, for RBC or schizonts, and for rings, respectively. In parallel with the ring and schizont transfections, RBCs were electroporated as in B using pulse CM-150 and buffer SE. [file 1475-2875-11-22-S3.PDF]
