# Supplementary figures and images for: Plate-based transfection and culturing technique for genetic manipulation of Plasmodium falciparum
Source: Malar J. 2012 Jan 18;11:22. doi: 10.1186/1475-2875-11-22 (PMC3293776; doi:10.1186/1475-2875-11-22)

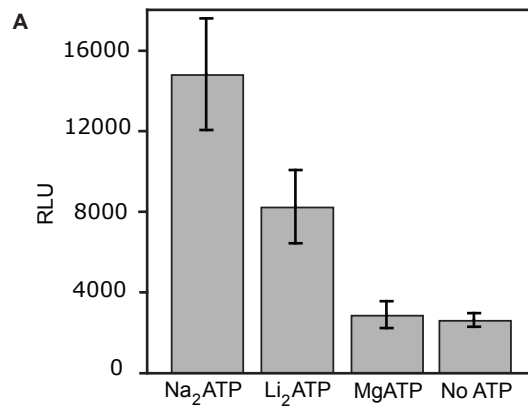

**B**

|       |     | μl RBC                      |       |       |        |       |       |
|-------|-----|-----------------------------|-------|-------|--------|-------|-------|
|       |     | 2                           | 4     | 6     | 2      | 4     | 6     |
| μgDNA | 2.5 | 19216                       | 43095 | 20285 | 11242  | 15262 | 19632 |
|       | 5   | 26961                       | 24761 | 31437 | 10071  | 33711 | 17293 |
|       | 10  | 50197                       | 39195 | 41491 | 12708  | N/A   | 20958 |
|       | 10  | 48371                       | 46838 | 55902 | 11918  | 16644 | 19722 |
|       |     | 103559                      | 83111 | 97289 | 48466  | 52859 | 58154 |
|       |     | 50067                       | 89251 | 77208 | 41206  | 18746 | 56843 |
|       |     | 6.25 mM Li <sub>2</sub> ATP |       |       | No ATP |       |       |

Supplement: Additional file 4 — Optimization transfection mixture components. (A) Transfection efficiency using different ATP salts. 6 μl packed RBCs were transfected (pulse CM-162) with 5 μg of the RLUC reporter plasmid, in Buffer SE and 6.25 mM final concentration of different ATP salts (Na2ATP, Li2ATP and MgATP). RLUC reporter signal was measured 48 h later for six replicas of each condition. Error bars, s.e.m. (B) Transfection efficiency using different volumes of packed RBCs. Three different volumes (2, 4 and 6 μl) of packed RBCs were transfected (pulse CM-162) with 2.5, 5 or 10 μg RLUC reporter plasmid, in Buffer SE with or without 10 mM Li2ATP. Table shows RLU values measured 48 h later for two replicas of each condition. [file 1475-2875-11-22-S4.PDF]

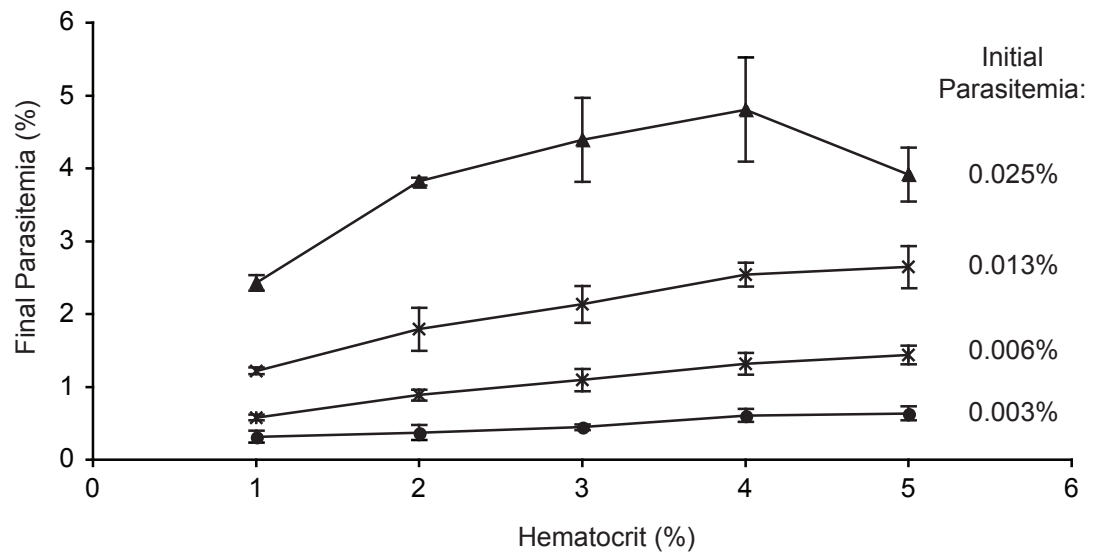

Supplement: Additional file 5 — Optimization of hematocrit for culture in 96-well plates. 200 μl of 0.025, 0.013, 0.006, 0.003% parasitaemia cultures (ring stage) were plated at 1, 2, 3, 4, and 5% HC in a 96-well flat-bottom plate, in triplicate. Five days later parasitaemia was measured by mMSF assay for each condition. Error bars, standard deviation. [file 1475-2875-11-22-S5.PDF]

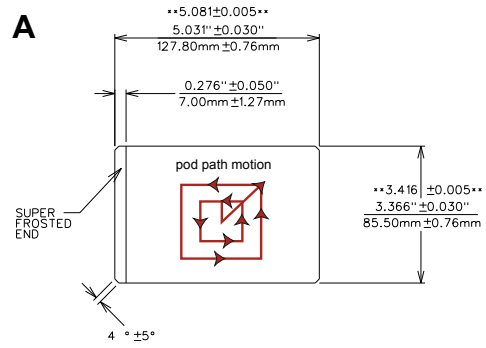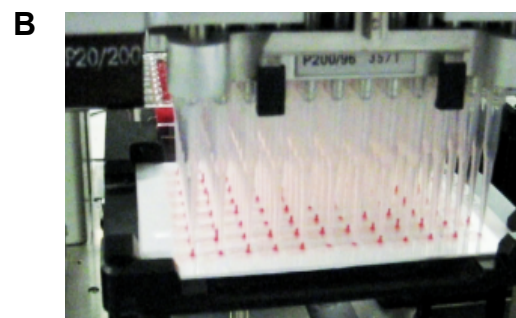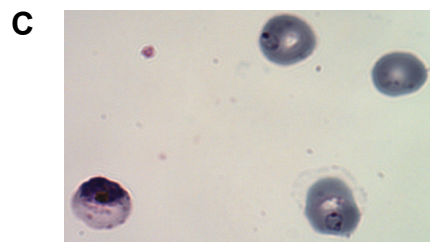

Supplement: Additional file 7 — Automated blood smearing. (A) Custom slide dimensions. (B) Snapshot of the automated blood smearing process. The Beckman 96-channel BioMek-NX liquid handler dispenses 1 μl of culture at 0.1 mm above the slide surface as the tips trace two concentric 0.33 and 0.66 mm wide squares. (D) Representative Giemsa-stain of one of the 96 smears on a slide [19]. [file 1475-2875-11-22-S7.PDF]
