# Supplementary material for: Plate-based transfection and culturing technique for genetic manipulation of Plasmodium falciparum
Source: Malar J. 2012 Jan 18;11:22. doi: 10.1186/1475-2875-11-22 (PMC3293776; doi:10.1186/1475-2875-11-22)
Supplement: Additional file 6 — Modified MSF assay. (A) For each tested condition, 200 μl of 0.8% parasitaemia cultures at 2% HC, were set up in columns 1 to 10 of five 96-well plates, uninfected RBCs were plated in column 11 for background signal measurement, and column 12 was used to generate a standard curve for parasitaemia. Final parasitaemia was measured 72 h later by MSF assay using five different manipulations before the measurement; media change, spent media was replaced with fresh media; no media change, culture was resuspended in the spent media; media wash/PBS resuspend, spent media was replaced with fresh media and culture was washed with 1X PBS; PBS resuspend, spent media was replaced with 1X PBS; PBS washed, spent media was replaced with 1X PBS and further washed in PBS. (B) For each of the five manipulations the mean of 10 column measurements (n = 80), their corresponding mean background signals (n = 8) and signal to background ratios, are listed. (C) Standard curve obtained using the PBS resuspend manipulation. Note that parasitaemias as low as 0.5% can be detected. [file 1475-2875-11-22-S6.PDF]

A

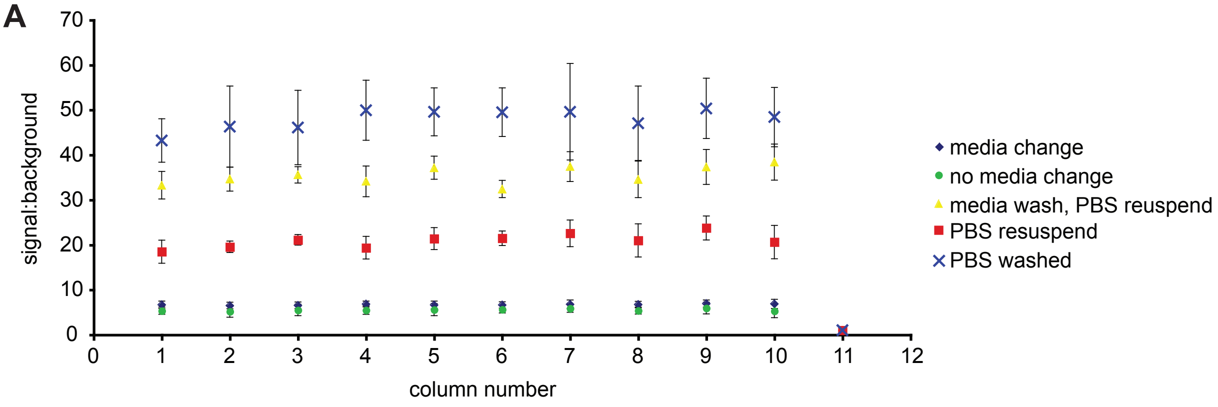

B

	mean signal*	mean bckgd	signal:bckgd
media change	4.03×10^6	6.08×10^5	6.63
No media change	2.73×10^6	5.41×10^5	5.05
Resuspend in PBS	2.82×10^6	1.35×10^5	20.92
Media wash/Resuspend in PBS	3.17×10^6	1.27×10^5	24.84
PBS washed	2.21×10^6	4.61×10^4	47.99

*Values for 4% parasitemia

C

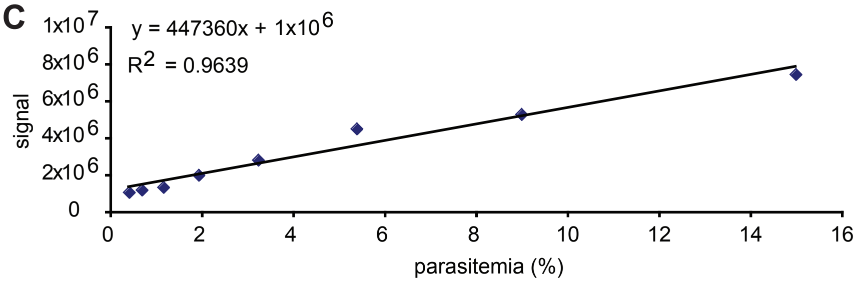

**parasitemia %**	**mean signal**
15.00	7416921
9.00	5251223
5.40	4470397
3.24	2781849
1.94	1970950
1.17	1305287
0.70	1161885
0.42	1040855
